# Higher Dietary Phytochemical Index Is Associated With Lower Disease Activity, Inflammation, and Gut Dysbiosis in Patients With Ulcerative Colitis

**DOI:** 10.1002/fsn3.71134

**Published:** 2025-10-30

**Authors:** Yue Zhu, Zhihui Lu, Jian Quan, Lingmin Wu

**Affiliations:** ^1^ Department of Oncology 302 Hospital of China Guizhou Aviation Industry Group Anshun City Guizhou Province China; ^2^ Department of Gastroenterology 302 Hospital of China Guizhou Aviation Industry Group Anshun City Guizhou Province China; ^3^ Clinical Laboratory 302 Hospital of China Guizhou Aviation Industry Group Anshun City Guizhou Province China

**Keywords:** dietary phytochemicals, gut microbiota, inflammation, quality of life, ulcerative colitis

## Abstract

Dietary phytochemicals possess anti‐inflammatory and antioxidant properties that may modulate disease activity in ulcerative colitis (UC). This study examined the association between the Dietary Phytochemical Index (DPI) and disease activity, biomarkers, gut microbiota, and psychological health in UC patients. In this cross‐sectional study of 350 UC patients, dietary intake was assessed using a validated FFQ to calculate the DPI. Participants were stratified into DPI quartiles. Disease activity was measured via the Mayo score. Biomarkers (FCP, CRP, IL‐6, ESR, homocysteine, zonulin), gut microbiota diversity, Firmicutes/Bacteroidetes ratio, and SCFA levels were analyzed. Psychological status and quality of life were evaluated using validated questionnaires (PHQ‐9, GAD‐7, PSQI, FSS, SIBDQ, IBD Disability Index). Participants with higher DPI scores showed significantly lower levels of FCP, CRP, IL‐6, ESR, homocysteine, and zonulin (all *p* < 0.001). Gut microbial richness (Shannon index) and the F/B ratio increased with higher DPI quartiles (all *p* < 0.001), indicating reduced inflammation (all *p* < 0.001). In terms of SCFAs, butyrate levels were significantly higher in Q4 (94.83 μmol/g) compared to Q1 (68.30 μmol/g, *p* < 0.001). In healthy adults, fecal butyrate concentrations typically range between 60 and 100 μmol/g; thus, the observed increase in Q4 reflects a clinically meaningful improvement. Q4 participants reported significantly better sleep quality, lower depression and anxiety scores, reduced fatigue and disability, and higher quality of life (all *p* < 0.001). The Mayo score, reflecting clinical disease activity, was significantly lower in Q4 (4.90 ± 0.83) compared to Q1 (6.18 ± 1.95, *p* < 0.001). Higher DPI adherence was associated with lower disease activity and improved clinical outcomes, though causal relationships cannot be inferred due to the cross‐sectional design.

## Graphical abstract

A higher dietary phytochemical index was significantly associated with lower disease activity, reduced systemic and intestinal inflammation, and improved gut microbiota diversity in ulcerative colitis patients. These findings suggest that phytochemical‐rich diets may offer a supportive strategy to improve clinical outcomes and gut health in UC management.

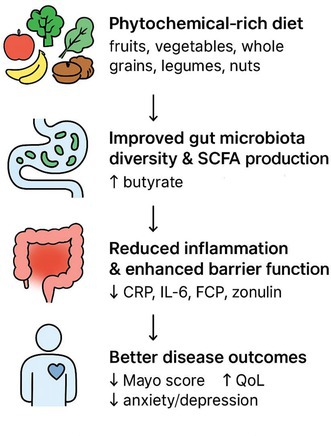

## 1 Introduction

Ulcerative colitis (UC) is a chronic inflammatory bowel disease characterized by relapsing–remitting inflammation of the colon, leading to significant health burden and reduced quality of life (Segal et al. 2021; Preda and Istrătescu 2022; Du and Ha 2020; Guo et al. 2020). It arises from genetic, immune, environmental, and microbial factors, with inflammation and gut dysbiosis playing key roles in disease progression (Segal et al. 2021; Preda and Istrătescu 2022; Du and Ha 2020; Guo et al. 2020). Consequently, identifying modifiable lifestyle factors that can favorably influence these pathways has become a major focus of research.

Diet is increasingly recognized as a pivotal environmental determinant influencing both the gut microbiome composition and systemic inflammation in UC (Keshteli et al. 2019; Talebi et al. 2023; Mousavi et al. 2022). In particular, diets rich in phytochemicals—bioactive compounds predominantly found in plant‐based foods such as fruits, vegetables, whole grains, nuts, and legumes—have garnered attention due to their potent antioxidant, anti‐inflammatory, and immunomodulatory properties (Keshteli et al. 2019; Halmos et al. 2024; Jowett et al. 2004; Amirkhizi et al. 2023). These compounds include polyphenols, flavonoids, carotenoids, and other plant secondary metabolites, which can modulate oxidative stress and immune responses while fostering beneficial microbial communities (Saxena et al. 2014). Polyphenols exert anti‐inflammatory effects through multiple mechanisms, including inhibition of NF‐κB signaling, modulation of cytokine profiles, enhancement of SCFA production by gut microbes, and reinforcement of epithelial barrier integrity. These properties are particularly relevant in the context of UC, where mucosal inflammation and barrier dysfunction are central to pathogenesis (Del Bo et al. 2021).

Previous studies have demonstrated that higher intake of plant‐based foods and specific dietary patterns and indices—such as the low inflammatory diet index (DII)—are associated with reduced inflammation and improved clinical outcomes in inflammatory bowel disease (IBD) patients, including those with UC (Nikniaz et al. 2024; Gu et al. 2025; DuBois et al. 2024). For instance, several observational studies have linked polyphenol‐rich diets to decreased circulating inflammatory markers such as TNF‐α and CRP (Caban et al. 2024). Additionally, other research has reported favorable shifts in gut microbiota composition, including a decreased abundance of beneficial bacteria, changes in the Firmicutes to Bacteroidetes (F/B) ratio, and improvements in alpha diversity measured by the Shannon index, along with reductions in opportunistic pathogens (Shen et al. 2018). However, many of these studies focus either on isolated phytochemicals or broad dietary patterns, without providing a comprehensive measure of total dietary phytochemical exposure.

The Dietary Phytochemical Index (DPI) has emerged as a valuable tool to estimate overall dietary phytochemical intake, reflecting the proportion of energy derived from phytochemical‐rich foods (Qorbani et al. 2022). Unlike other indices such as the Mediterranean Diet Score or the DII, which assess broader dietary patterns or inflammation potential, the DPI specifically quantifies phytochemical‐rich food intake, which may have more direct implications for gut microbiota modulation and immune regulation in UC. Recent studies have demonstrated inverse associations between higher DPI and biomarkers of inflammation, oxidative stress, and chronic disease risk (Kim and Park 2022; Mehranfar et al. 2022). However, few investigations have assessed these relationships specifically in UC patients, where dietary phytochemicals may modulate immune responses, oxidative damage, and microbial dysbiosis. Despite its growing application in chronic disease research, limited data exist on the association between DPI and UC disease activity, inflammatory biomarkers, and gut microbial dysbiosis. Most existing studies lack integrated analyses that connect dietary phytochemical intake with both inflammatory status and gut microbiota profiles in UC patients. This study builds on prior research by applying the DPI to UC patients, integrating assessments of diet, inflammation, gut microbiota, and psychological outcomes. While previous studies have explored phytochemicals in IBD (Talebi et al. 2023; Nikniaz et al. 2024; Gu et al. 2025; DuBois et al. 2024; Morvaridi et al. 2020; Touny et al. 2024), few have used DPI to comprehensively evaluate its associations with clinical, microbial, and psychological parameters in UC, contributing to a more holistic understanding of dietary impacts.

To address this gap, we evaluated the relationship between DPI and clinical disease activity, systemic inflammation, and gut microbial composition in patients with UC. This study aims to investigate the associations of DPI with clinical disease activity, inflammatory biomarkers, and gut microbiota profiles in patients with ulcerative colitis, thereby addressing the gap in understanding the holistic impact of dietary phytochemicals in modulating UC pathophysiology. These findings could have important implications for developing dietary interventions aimed at reducing inflammation and restoring gut microbial balance, thereby complementing existing pharmacological treatments and improving patient outcomes.

## 2 Methods

### 2.1 Study Design and Participants

**FIGURE 1:**
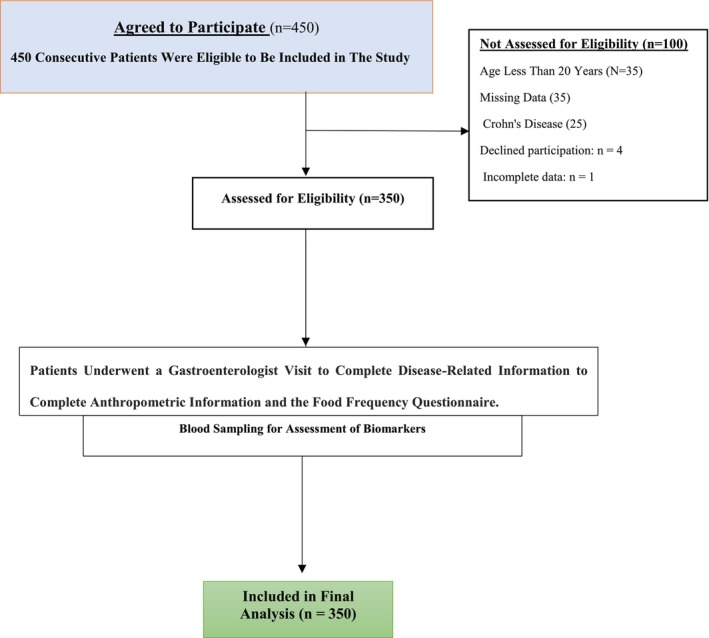
Flowchart illustrating the selection of 350 ulcerative colitis patients, detailing recruitment, screening, inclusion/exclusion criteria, and final cohort for gut microbiota and inflammatory marker analysis.

This analytical cross‐sectional study was conducted between February 2023 and April 2024 at the Department of Gastroenterology, 302 Hospital of China Guizhou, Anshun City, Guizhou Province, China. A total of 450 consecutive patients referred to the Gastroenterology clinic were assessed for eligibility. After excluding four patients who declined to participate and three with incomplete information, data from 350 UC patients were included in the final analysis (Figure 1). This cross‐sectional investigation was conducted in accordance with the STROBE guidelines. Ethical approval was granted by the Ethics Committee of 302 Hospital of China Guizhou Aviation Industry Group, Anshun, China (Approval Code: 20221105), and written informed consent was obtained from all participants prior to enrollment.

Participants were eligible if they were adults aged 20–60 years with a confirmed diagnosis of ulcerative colitis (UC) for at least 6 months prior to study entry. The diagnosis was established by an expert gastroenterologist based on clinical symptoms (e.g., chronic diarrhea, rectal bleeding, abdominal pain), endoscopic findings (e.g., continuous mucosal inflammation, ulceration, or erythema assessed via colonoscopy), and histological confirmation of chronic inflammation (e.g., crypt distortion, inflammatory cell infiltration), in accordance with the European Crohn's and Colitis Organization (ECCO)–European Society of Gastrointestinal and Abdominal Radiology (ESGAR) guidelines (Maaser et al. 2019). Patients were excluded if they had other gastrointestinal disorders (e.g., Crohn's disease, irritable bowel syndrome, infectious colitis), malignancies (e.g., colorectal cancer), autoimmune diseases (e.g., rheumatoid arthritis, systemic lupus erythematosus), or comorbidities requiring specific dietary interventions (e.g., type 1 or type 2 diabetes mellitus, cardiovascular disease, celiac disease, chronic kidney disease). Additionally, pregnant or lactating women, individuals under 20 or over 60 years of age, those with a UC diagnosis less than 6 months prior, or those with incomplete clinical or dietary data were excluded to ensure a homogeneous study population and minimize confounding.

### 2.2 Sample Size Determination

The sample size was determined using PASS software (version 2021; NCSS, USA), ensuring 80% statistical power and a 95% confidence level. The calculation was informed by prior research examining the relationship between dietary intake and relapse risk in individuals with UC (Bakhtiari et al. 2024).

### 2.3 Sociodemographic and Clinical Assessments

Sociodemographic characteristics were collected using a structured questionnaire and included age, sex, education level (illiterate, sub‐diploma, diploma, and university education), marital status (married, single), smoking status (yes/no), and alcohol consumption (yes/no). Disease‐related characteristics such as disease duration (years), disease extent, medication use (mesalazine, azathioprine, prednisolone), and supplement intake (vitamins, minerals, omega‐3) were recorded by an expert gastroenterologist. The anatomical extent of UC was classified according to the Montreal classification system into three categories: E1 (ulcerative proctitis, involving only the rectum), E2 (left‐sided colitis, involving the rectum and a portion of the colon up to the splenic flexure), and E3 (extensive colitis or pancolitis, involving areas beyond the splenic flexure, possibly including the entire colon). Disease extension was determined based on colonoscopy findings and physician documentation.

Disease activity was assessed using the Mayo score, which incorporates four components: stool frequency, physician's global assessment, rectal bleeding, and endoscopic findings. Each component is scored from 0 to 3 (endoscopy up to 12), yielding a total score ranging from 0 to 12. A higher Mayo score indicates more severe disease activity (Lewis et al. 2008).

Physical activity was assessed using the validated International Physical Activity Questionnaire (IPAQ‐short form), and total MET‐minutes per week was calculated as a continuous variable (Lee et al. 2011).

### 2.4 Dietary Assessment and Phytochemical Index Calculation

Participants' dietary intake over the preceding year was assessed using a validated 149‐item semi‐quantitative food frequency questionnaire (FFQ), administered through face‐to‐face interviews by trained nutrition professionals. Respondents reported the frequency and portion sizes of consumed foods, which were standardized using household measures (e.g., cups, spoons) and visual aids (e.g., food models) to enhance accuracy and minimize recall bias. Dietary data were converted into average daily intake values using Nutritionist IV (version 4.0, N‐Squared Computing, USA), validated for the Chinese population. Recall over a year remains a limitation, particularly in UC patients with fluctuating symptoms, which may affect reporting accuracy.

The DPI was computed as the proportion of total daily energy intake obtained from foods rich in phytochemicals, using the following formula:

$$\mathrm{DPI}\;\left(\%\right)=\left(\text{Energy from phytochemical}-\text{rich foods}/\text{Total daily energy intake}\right)\times 100.$$

Phytochemical‐rich foods included fruits, vegetables (excluding potatoes due to their low phytochemical density), legumes, whole grains, nuts, seeds, soy products, and olive oil. Foods low in phytochemicals (refined grains, animal products, sweets, and added fats) were excluded from the numerator (Qorbani et al. 2022). Participants were stratified into quartiles (Q1–Q4) based on DPI scores.

### 2.5 Inflammatory and Microbiota Biomarkers

Biomarkers assessed included fecal calprotectin (FCP), C‐reactive protein (CRP), interleukin‐6 (IL‐6), erythrocyte sedimentation rate (ESR), homocysteine, and zonulin. Biomarkers were selected based on their validated roles in monitoring UC inflammation: FCP for intestinal inflammation, CRP and IL‐6 for systemic responses, ESR for inflammatory burden, homocysteine for metabolic stress, and zonulin for gut permeability. Serum CRP was measured using a high‐sensitivity immunoassay (Roche Diagnostics, Mannheim, Germany) and reported in mg/L. ESR, another marker of systemic inflammatory burden, was determined via the Westergren method and expressed in mm/h. IL‐6 levels were quantified using high‐sensitivity ELISA kits (R&D Systems, Minneapolis, USA) to reflect pro‐inflammatory cytokine activity, while C3 concentrations were measured by immunoturbidimetric assay (Siemens Healthineers, Erlangen, Germany) as an indicator of immune system activation. Homocysteine, a marker of metabolic stress and vascular inflammation, was measured enzymatically (Diazyme Laboratories, Poway, USA). Zonulin, a protein regulating intestinal tight junctions and serving as a proxy for intestinal permeability, was determined in serum via ELISA (Immundiagnostik, Bensheim, Germany) and reported in ng/mL.

### 2.6 Gut Microbiota

Stool DNA was extracted (QIAamp DNA Stool Mini Kit, Qiagen). The 16S rRNA V3–V4 region was amplified and sequenced on Illumina MiSeq. Raw data were processed with QIIME2 (version 2022.2). Alpha diversity indices (Shannon, Simpson) and Firmicutes/Bacteroidetes ratio were computed. Additionally, fecal calprotectin (FCP) was measured using an ELISA kit (Wiz Biotech, Xiamen, China).

To further investigate gut microbial activity, levels of short‐chain fatty acids (SCFAs)—including butyrate, propionate, acetate, and total SCFAs—were quantified in stool samples using gas chromatography. Gas chromatography was performed using an Agilent 7890B system equipped with an HP‐FFAP column (30 m × 0.25 mm × 0.25 μm) and a flame ionization detector (FID) for SCFA quantification, ensuring reproducible measurements. These microbial metabolites are products of dietary fiber fermentation by gut bacteria and are known for their anti‐inflammatory, immunomodulatory, and gut‐barrier protective effects. SCFA concentrations were reported in micromoles per gram (μmol/g) of fecal matter.

### 2.7 Psychological Assessment

In addition to biological measures, several psychological, behavioral, and quality‐of‐life dimensions were assessed using validated patient‐reported outcome measures. Sleep quality was assessed by the Pittsburgh Sleep Quality Index (PSQI), which assesses seven domains of sleep over the previous month; scores range from 0 to 21, with higher scores indicating poorer sleep quality, and a cutoff score > 5 denoting poor sleep (Hood et al. 2018).

Depressive symptomatology was evaluated employing the Patient Health Questionnaire‐9 (PHQ‐9), a standardized tool in which each of the nine criteria is rated on a scale from 0 (“not at all”) to 3 (“nearly every day”), yielding an overall score between 0 and 27 (Litster et al. 2018).

To assess chronic fatigue, the Fatigue Severity Scale (FSS) was administered, consisting of nine items scored on a 7‐point Likert scale; higher mean scores indicate greater fatigue severity. Anxiety signs were assessed by the Generalized Anxiety Disorder‐7 (GAD‐7) scale, with a similar scoring structure and a maximum score of 21 (Bernstein et al. 2018).

The IBD Disability Index was used to evaluate the functional impact of disease on daily activities, work productivity, and social participation, offering a multidimensional understanding of disease burden. Finally, disease‐specific health‐related quality of life (HRQoL) was evaluated using the Short Inflammatory Bowel Disease Questionnaire (SIBDQ), a validated instrument comprising 10 questions organized into four subscales: bowel symptoms, emotional well‐being, systemic manifestations, and social functioning. Overall scores range from 10 to 70, with increasing values reflecting improved quality of life (Chen et al. 2017).

### 2.8 Statistical Analysis

The normality of continuous variables was evaluated through the K‐S test. Continuous outcomes were summarized using means and standard deviations (SD), whereas categorical data were presented as counts and corresponding percentages. To compare differences across DPI quartiles, one‐way analysis of variance (ANOVA) was applied for normally distributed data, while the Kruskal–Wallis H test was used for non‐normal distributions. Categorical variables were analyzed using the chi‐square test. The relationship between DPI and the continuous Mayo score outcome was examined via linear regression analysis. Post hoc analyses were performed using Tukey's HSD for ANOVA and Bonferroni correction for Kruskal–Wallis tests. Multicollinearity in multivariable models was assessed via variance inflation factor (VIF), with all covariates showing VIF < 2. Alcohol consumption and smoking were initially considered but were non‐significant and not included in the final model. Statistical analyses were performed using IBM SPSS Statistics for Windows, Version 21.0 (IBM Corp., Armonk, NY, USA). A *p*‐value less than 0.05 was considered indicative of statistical significance.

## 3 Results

Table 1 presents the baseline demographic, clinical, and dietary characteristics of the study participants stratified by quartiles of the DPI. The mean DPI score for the total cohort (*N* = 350) was 33.13 ± 3.90, with significant differences observed across quartiles (*p* < 0.001). There were no statistically significant differences in age, weight, BMI, sex distribution, marital status, education level, physical activity, smoking status, alcohol consumption, disease extension, Hemoglobin, use of nutritional supplements, or treatments (azathioprine, prednisolone, mesalazine) among the DPI quartiles (all *p* > 0.05). Albumin levels varied significantly across quartiles (*p* < 0.001), with higher DPI quartiles exhibiting higher albumin concentrations. Dietary intake of energy, fiber, carbohydrates, protein, and fat showed no significant differences across quartiles. Significant differences in magnesium and dietary fatty acid intake were observed across DPI quartiles. Participants in the highest quartile (Q4) had greater magnesium intake (352.21 ± 81.31 mg/day) than those in Q1 (279.27 ± 85.55 mg/day, *p* < 0.001). PUFA, MUFA, and omega‐3 intakes also increased with higher DPI scores (e.g., PUFA Q4: 19.07 ± 0.56 g/day; omega‐3 Q4: 1.91 ± 0.06 g/day; all *p* < 0.001), while SFA intake was significantly lower (Q4: 10.19 ± 4.91 g/day), indicating a healthier fat profile among those with greater phytochemical‐rich diet adherence.

**TABLE 1:** Baseline demographic, clinical, and dietary characteristics of participants by DPI quartiles.

Variables	Total (*N* = 350)	Q1 (*N* = 92)	Q2 (*N* = 87)	Q3 (*N* = 93)	Q4 (*N* = 78)	*p*
DPI (mean)	33.13 ± 3.90	28.36 ± 0.72	31.36 ± 1.22	35.00 ± 0.85	38.50 ± 1.27	< 0.001
Age (years)	41.13 ± 4.97	41.01 ± 6.45	40.36 ± 3.44	41.92 ± 5.21	41.18 ± 3.92	0.208
Weight (kg)	70.54 ± 5.98	69.12 ± 6.05	71.28 ± 5.29	70.87 ± 5.03	70.97 ± 7.36	0.064
BMI	24.14 ± 0.93	24.30 ± 1.33	24.09 ± 0.59	24.14 ± 0.72	24.00 ± 0.89	0.180
Sex, Female, *n* (%)	178 (50.9)	48 (13.7)	46 (13.1)	47 (13.4)	37 (10.6)	0.902
Marital Status, *n* (%)						
Married	179 (51)	46 (50)	34 (39)	54 (58)	45 (57)	0.191
Single	171 (49)	46 (60)	53 (41)	39 (42)	33 (42.3)	
Education Level, *n* (%)						
Illiterate	24 (6.9)	6 (6.5)	8 (9.2)	4 (4.3)	6 (7.7)	0.417
Under Diploma	89 (25.5)	27 (29.3)	21 (24.1)	28 (30)	13 (16.7)	
Diploma	87 (25)	27 (29.3)	20 (23.0)	21 (22.7)	19 (24.4)	
College	150 (42.9)	32 (34.8)	38 (43.7)	40 (43.0)	40 (51.3)	
Physical Activity (total MET)	575.8 ± 69.42	574.14 ± 90.56	564.03 ± 48.58	586.72 ± 48.99	576.13 ± 54.51	0.680
Smoking, *n* (%)	113 (32.3)	25 (7.1)	34 (9.7)	27 (7.7)	27 (7.7)	0.417
Alcohol, *n* (%)	79 (22)	21 (22)	20 (23)	26 (28)	12 (15.5)	0.277
Disease extension						
E1	24 (6.9)	6 (6.5)	6 (6.9)	4 (4.3)	8 (10.3)	0.680
E2	239 (68.3)	59 (64.1)	61 (70.1)	68 (73.1)	51 (56.5)	
E3	87 (24.9)	27 (29.3)	20 (23)	21 (22.6)	19 (24.4)	
Nutritional Supplements, *n* (%)	212 (60.5)	56 (61)	52 (60)	55 (59)	49 (63)	0.985
Treatments, *n* (%)						
Azathioprine, *n* (%)	274 (81.5)	75 (81)	66 (75)	75 (80)	58 (75)	0.598
Prednisolone, *n* (%)	105 (30)	28 (30.5)	23 (26.5)	32 (34.5)	22 (28.2)	0.679
Mesalazine, *n* (%)	350 (100)	92 (100)	87 (100)	93 (100)	78 (100)	—
Hemoglobin (g/dL)	12.8 ± 2.0	12.96 ± 2.5	13.19 ± 2.1	12.4 ± 1.44	12.37 ± 1.16	0.070
Albumin (g/dL)	4.4 ± 1.82	3.54 ± 1.64	3.84 ± 1.04	4.16 ± 1.95	4.73 ± 1.26	< 0.001
Energy (kcal/day)	2107.8 ± 321.99	2095.1 ± 341.04	2093.71 ± 285.4	2137.9 ± 335.06	2102.6 ± 325.29	0.769
Fiber (g/day)	20.42 ± 14.75	18.39 ± 14.31	20.26 ± 7.85	19.76 ± 17.52	23.77 ± 17.08	0.113
Carbohydrates (g/day)	276.69 ± 69.95	275.25 ± 76.69	266.29 ± 57.10	286.91 ± 61.92	277.85 ± 82.32	0.266
Protein (g/day)	69.45 ± 16.38	68.43 ± 17.41	70.30 ± 15.99	69.48 ± 15.85	69.69 ± 16.44	0.896
Fat (g/day)	70.64 ± 15.82	70.90 ± 14.48	73.62 ± 14.90	69.05 ± 16.35	68.91 ± 17.42	0.174
Magnesium (mg/day)	310.39 ± 78.13	279.27 ± 85.55	274.07 ± 54.19	340.52 ± 55.19	352.21 ± 81.31	< 0.001
PUFA (g/day)	16.93 ± 2.25	14.79 ± 0.93	15.82 ± 1.87	18.30 ± 1.83	19.07 ± 0.56	< 0.001
MUFA (g/day)	13.93 ± 2.31	11.90 ± 1.42	12.82 ± 1.87	15.30 ± 1.83	15.97 ± 1.05	< 0.001
Omega‐3 fatty acids (g/day)	1.69 ± 0.23	1.48 ± 0.09	1.58 ± 0.19	1.83 ± 0.18	1.91 ± 0.06	< 0.001
Saturated Fatty Acids (g/day)	14.93 ± 3.44	16.61 ± 0.38	16.20 ± 0.54	16.02 ± 0.35	10.19 ± 4.91	< 0.001
Disease Duration (months)	6.40 ± 2.04	6.21 ± 1.71	6.63 ± 2.48	6.12 ± 1.59	6.71 ± 2.26	0.139

*Note:* Data are presented as mean ± standard deviation (SD) for continuous variables and *n* (%) for categorical variables. *p* values denote the significance of differences across quartiles (Q1, Q2, Q3, Q4). Statistical methods: One‐way ANOVA was used for continuous variables, and chi‐square tests were applied for categorical variables to assess differences across DPI quartiles.

Abbreviations: BMI, body mass index; DPI, Dietary Phytochemical Index; F/B, Firmicutes‐to‐Bacteroidetes ratio; MET, metabolic equivalent of task; SCFA, short‐chain fatty acids.

As shown in Table 2, the F/B ratio increased progressively with higher DPI quartiles (*p* < 0.001), indicating a significant association between the DPI index and gut microbiota composition. Measures of microbial diversity, including Shannon and Simpson indices, were significantly higher in participants with greater DPI scores (*p* < 0.001), suggesting enhanced microbial richness and evenness. Inflammatory biomarkers, including FCP, CRP, interleukin‐6 (IL‐6), ESR, and homocysteine, showed a significant decreasing trend with increasing DPI quartiles (all *p* < 0.001), consistent with reduced systemic and intestinal inflammation among participants with higher DPI scores. Additionally, SCFAs, including butyrate, propionate, acetate, and total SCFAs, were significantly elevated in higher DPI quartiles (*p* < 0.001), indicating favorable microbial metabolic activity. These increases in SCFA levels (e.g., butyrate from 68.30 to 94.83 μmol/g) exceed typical thresholds for clinical relevance in UC, where levels below 70 μmol/g are associated with active disease (Kaczmarczyk et al. 2021).

**TABLE 2:** Distribution of inflammatory biomarkers and gut microbiome diversity indices across DPI quartiles.

Variables	Total (*N* = 350)	Q1 (*N* = 92)	Q2 (*N* = 87)	Q3 (*N* = 93)	Q4 (*N* = 78)	*p*	*p*‐trend
Firmicutes/Bacteroidetes Ratio	1.95 ± 0.44	1.49 ± 0.10	1.76 ± 0.19	2.05 ± 0.22	2.57 ± 0.26	< 0.001	< 0.001
Shannon Index	3.81 ± 0.33	3.46 ± 0.12	3.68 ± 0.14	3.89 ± 0.15	4.28 ± 0.15	< 0.001	< 0.001
Simpson Index	0.56 ± 0.04	0.51 ± 0.02	0.55 ± 0.02	0.58 ± 0.02	0.62 ± 0.01	< 0.001	< 0.001
FCP (μg/g)	125.36 ± 17.01	144.27 ± 8.59	130.86 ± 8.68	120.18 ± 9.51	103.17 ± 6.11	< 0.001	< 0.001
CRP (mg/L)	4.03 ± 1.16	5.44 ± 0.61	4.18 ± 0.63	3.67 ± 0.60	2.60 ± 0.39	< 0.001	< 0.001
IL‐6 (pg/mL)	2.54 ± 0.70	3.44 ± 0.36	2.64 ± 0.34	2.20 ± 0.35	1.75 ± 0.17	< 0.001	< 0.001
C3 (mg/dL)	108.21 ± 21.98	88.54 ± 7.38	94.40 ± 6.57	112.40 ± 13.00	141.67 ± 6.28	< 0.001	< 0.001
ESR (mm/h)	18.76 ± 8.31	27.70 ± 3.52	22.93 ± 3.67	15.38 ± 4.79	7.67 ± 1.26	< 0.001	< 0.001
Homocysteine (μmol/L)	11.49 ± 2.68	12.53 ± 1.82	12.05 ± 0.30	12.16 ± 0.70	8.86 ± 4.35	< 0.001	< 0.001
SCFA Butyrate (μmol/g)	78.05 ± 11.37	68.30 ± 4.37	71.48 ± 3.90	79.68 ± 8.40	94.83 ± 3.26	< 0.001	< 0.001
SCFA Propionate (μmol/g)	195.34 ± 21.47	176.47 ± 8.76	183.66 ± 8.79	199.95 ± 16.36	225.00 ± 9.64	< 0.001	< 0.001
Acetate (μmol/g)	245.34 ± 21.47	226.47 ± 8.76	233.66 ± 8.79	249.95 ± 16.36	275.00 ± 9.64	< 0.001	< 0.001
SCFA Total (μmol/g)	518.73 ± 54.09	471.24 ± 21.88	488.80 ± 21.46	529.57 ± 40.54	594.83 ± 21.91	< 0.001	< 0.001
Zonulin (ng/mL)	227.62 ± 85.83	245.28 ± 96.26	229.79 ± 57.10	235.86 ± 75.80	194.55 ± 101.79	0.001	0.002

*Note:* Data are presented as mean ± standard deviation (SD). *p* values indicate the significance of differences across quartiles (Q1, Q2, Q3, Q4). Statistical methods: One‐way ANOVA was performed to compare mean values of biomarkers and diversity indices across DPI quartiles.

Abbreviations: C3, Complement Component 3; CRP, C‐Reactive Protein; ESR, Erythrocyte Sedimentation Rate; FCP, Fecal Calprotectin; IL‐6, Interleukin‐6; SCFA, Short‐Chain Fatty Acids.

Serum zonulin, an indicator of intestinal permeability, was lower in Q4 (194.55 ng/mL) than in Q1 (245.28 ng/mL), suggesting improved epithelial barrier function with higher DPI adherence (Table 2). These values approach the normal range for healthy adults (approximately 30–200 ng/mL (Artis 2008)), indicating a clinically relevant improvement in gut barrier integrity.

Table 3 summarizes the relationship between DPI quartiles and patient‐reported outcomes as well as clinical disease activity, with significant improvements observed in higher quartiles. Participants in the highest DPI quartile reported better sleep quality (mean PSQI: 4.50 vs. 14.39, *p* < 0.001). Quality of life, assessed by the SIBDQ, enhanced significantly with increasing DPI scores (*p* < 0.001). Psychological distress markers including anxiety (GAD‐7) and depression (PHQ‐9) scores were significantly reduced in higher DPI quartiles (both *p* < 0.001). Measures of disease‐related disability and fatigue (IBD Disability Index and Fatigue Severity Scale) also showed significant improvement with higher adherence to DPI (*p* < 0.001). Clinical disease activity, as reflected by the Mayo score, was significantly lower in participants with higher DPI quartiles (*p* < 0.001), indicating less severe disease activity.

**TABLE 3:** Quality of life, psychological status, and disease activity scores by DPI quartiles.

Variables	Total (*N* = 350)	Q1 (*N* = 92)	Q2 (*N* = 87)	Q3 (*N* = 93)	Q4 (*N* = 78)	*p*	*p* trend
PSQI	9.63 ± 4.23	14.39 ± 1.92	11.02 ± 2.24	7.94 ± 2.87	4.50 ± 1.39	< 0.001	< 0.001
QOL Assessment SIBDQ	50.34 ± 4.45	44.29 ± 1.34	49.48 ± 1.66	52.51 ± 1.24	55.83 ± 1.35	< 0.001	< 0.001
PHQ‐9	8.80 ± 3.66	12.90 ± 1.56	9.95 ± 2.23	7.43 ± 2.41	4.33 ± 1.11	< 0.001	< 0.001
GAD‐7	8.90 ± 3.45	11.98 ± 2.44	10.08 ± 2.42	7.70 ± 2.46	5.38 ± 2.46	< 0.001	< 0.001
IBD Disability Index	22.94 ± 9.37	27.66 ± 1.55	23.52 ± 2.53	20.09 ± 4.14	20.16 ± 7.94	< 0.001	< 0.001
Fatigue FSS	11.17 ± 3.30	12.44 ± 4.76	11.74 ± 3.41	10.54 ± 0.78	9.78 ± 2.01	< 0.001	< 0.001
Mayo Score	5.59 ± 1.47	6.18 ± 1.95	5.91 ± 1.73	5.29 ± 0.40	4.90 ± 0.83	< 0.001	< 0.001

*Note:* Data are presented as mean ± standard deviation (SD). P values denote the significance of differences across quartiles (Q1, Q2, Q3, Q4). Statistical methods: One‐way ANOVA was performed to compare mean values of biomarkers and diversity indices across DPI quartiles.

Multivariable linear regression models (Table 4) confirmed that higher DPI quartiles were associated with improved microbial and inflammatory outcomes, independent of confounders. The results demonstrated that, compared to the lowest quartile (Q1), higher DPI quartiles were significantly associated with a higher F/B ratio (Q4: *β* = 1.084, 95% CI: 1.023–1.144, *p* < 0.001). Similarly, alpha diversity measures such as Shannon and Simpson indices showed positive associations with increasing DPI quartiles (Shannon index Q4: *β* = 0.823, 95% CI: 0.781–0.866, *p* < 0.001), indicating greater microbial diversity in participants with higher phytochemical‐rich diet adherence. Conversely, inflammatory markers including fecal calprotectin (FCP), C‐reactive protein (CRP), interleukin‐6 (IL‐6), erythrocyte sedimentation rate (ESR), and homocysteine exhibited significant inverse relationships with DPI quartiles. For example, participants in the highest DPI quartile (Q4) had substantially lower FCP levels (*β* = −40.88, 95% CI: −43.39 to −38.36, *p* < 0.001) and CRP (*β* = −2.82, 95% CI: −2.99 to −2.65, *p* < 0.001) compared to those in Q1. Zonulin levels, a marker of intestinal permeability, were also significantly reduced in higher DPI quartiles (Q4: *β* = −54.25, 95% CI: −79.83 to −28.67, *p* < 0.001), suggesting improved gut barrier integrity among participants with a higher DPI score. These findings collectively indicate that higher adherence to a phytochemical‐rich diet is strongly linked to a healthier gut microbial profile and reduced systemic and intestinal inflammation.

**TABLE 4:** Multivariable linear regression of DPI quartiles and gut microbiota and inflammatory markers in adults with ulcerative colitis.

Variable	DPI quartile	Crude model	Model 1	Model 2	*p* for trend
F/B Ratio	Q1	Ref	Ref	Ref	
	Q2	0.276 (0.218–0.334)	0.274 (0.216–0.332)	0.274 (0.217–0.332)	
	Q3	0.575 (0.519–0.632)	0.577 (0.520–0.634)	0.577 (0.521–0.634)	
	Q4	1.091 (1.031–1.150)	1.084 (1.023–1.144)	1.084 (1.024–1.144)	< 0.001
Shannon Index	Q1	Ref	Ref	Ref	
	Q2	0.220 (0.178–0.261)	0.225 (0.184–0.266)	0.223 (0.181–0.264)	
	Q3	0.427 (0.387–0.468)	0.434 (0.394–0.474)	0.435 (0.395–0.475)	
	Q4	0.819 (0.777–0.862)	0.829 (0.787–0.871)	0.823 (0.781–0.866)	< 0.001
Simpson Index	Q1	Ref	Ref	Ref	
	Q2	0.039 (0.034–0.045)	0.040 (0.034–0.045)	0.039 (0.034–0.045)	
	Q3	0.069 (0.064–0.075)	0.070 (0.065–0.075)	0.070 (0.065–0.075)	
	Q4	0.111 (0.105–0.117)	0.112 (0.106–0.117)	0.110 (0.105–0.116)	< 0.001
CRP	Q1	Ref	Ref	Ref	
	Q2	−1.26 (−1.43 to −1.09)	−1.26 (−1.43 to −1.09)	−1.24 (−1.41 to −1.08)	
	Q3	−1.77 (−1.93 to −1.60)	−1.77 (−1.93 to −1.60)	−1.77 (−1.94 to −1.61)	
	Q4	−2.84 (−3.01 to −2.67)	−2.85 (−3.02 to −2.67)	−2.82 (−3.00 to −2.65)	< 0.001
IL‐6	Q1	Ref	Ref	Ref	
	Q2	−0.80 (−0.89 to −0.70)	−0.80 (−0.89 to −0.70)	−0.77 (−0.87 to −0.69)	
	Q3	−1.23 (−1.33 to −1.14)	−1.23 (−1.32 to −1.14)	−1.23 (−1.32 to −1.14)	
	Q4	−1.69 (−1.78 to −1.59)	−1.68 (−1.78 to −1.58)	−1.66 (−1.75 to −1.56)	< 0.001
FCP	Q1	Ref	Ref	Ref	
	Q2	−13.41 (−15.89 to −10.93)	−13.76 (−16.23 to −11.29)	−13.36 (−15.77 to −10.95)	
	Q3	−24.09 (−26.52 to −21.66)	−24.14 (−26.56 to −21.72)	−24.20 (−26.56 to −21.84)	
	Q4	−41.11 (−43.65 to −38.56)	−41.43 (−43.97 to −38.89)	−40.93 (−43.44 to −38.42)	< 0.001
ESR	Q1	Ref	Ref	Ref	
	Q2	−4.77 (−5.83 to −3.70)	−4.87 (−5.94 to −3.81)	−4.74 (−5.78 to −3.69)	
	Q3	−12.32 (−13.36 to −11.27)	−12.25 (−13.30 to −11.21)	−12.26 (−13.28 to −11.24)	
	Q4	−20.03 (−21.12 to −18.93)	−20.04 (−21.14 to −18.95)	−19.86 (−20.95 to −18.77)	< 0.001
Homocysteine (μmol/L)	Q1	Ref	Ref	Ref	
	Q2	−1.90 (−2.62 to −1.18)	−1.82 (−2.54 to −1.10)	−0.51 (−1.18 to 0.17)	
	Q3	−4.28 (−4.99 to −3.57)	−4.21 (−4.91 to −3.50)	−0.40 (−1.06 to 0.27)	
	Q4	−6.59 (−7.33 to −5.85)	−6.48 (−7.22 to −5.74)	−3.80 (−4.51 to −3.09)	0.021
Zonulin (ng/mL)	Q1	Ref	Ref	Ref	
	Q2	−17.37 (−42.15 to 7.42)	−17.37 (−42.15 to 7.42)	−18.71 (−43.31 to 5.89)	
	Q3	−11.75 (−36.12 to 12.62)	−11.75 (−36.12 to 12.62)	−13.60 (−37.73 to 10.54)	
	Q4	−54.25 (−79.83 to −28.67)	−54.25 (−79.83 to −28.67)	−56.27 (−81.97 to −30.57)	0.008

*Note:* Values represent *β* coefficients and 95% confidence intervals (CIs) from linear regression analyses comparing quartiles of the DPI, with Quartile 1 (Q1) as the reference. *p*‐trend values were calculated using linear regression with DPI as a continuous variable to assess the linear trend of the association with each outcome variable. Crude model is unadjusted. Model 1 adjusts for age, sex, and body mass index (BMI). Model 2 further adjusts for disease duration, supplement use, disease extent, energy intake (kcal/day), physical activity (total MET) and fiber intake (g/day).

Abbreviations: CRP, C‐reactive protein; ESR, erythrocyte sedimentation rate; F/B Ratio, Firmicutes to Bacteroidetes ratio; FCP, fecal calprotectin; IL‐6, Interleukin‐6.

Table 5 presents the adjusted associations between DPI quartiles and patient‐reported outcomes including quality of life, psychological status, and clinical disease activity. Higher DPI quartiles were significantly correlated with better sleep quality as indicated by lower PSQI scores (Q4: *β* = −9.85, 95% CI: −10.55 to −9.33, *p* < 0.001). Similarly, quality of life measured by SIBDQ improved with increasing DPI scores (Q4: *β* = 11.54, 95% CI: 11.11 to 11.98, *p* < 0.001). Markers of psychological distress, including depression (PHQ‐9) and anxiety (GAD‐7) scores, decreased significantly across DPI quartiles, with the highest quartile showing the greatest reductions (PHQ‐9 Q4: *β* = −8.25, 95% CI: −9.01 to −7.90, *p* < 0.001; GAD‐7 Q4: *β* = −6.45, 95% CI: −7.19 to −5.71, *p* < 0.001). Measures of fatigue (Fatigue Severity Scale) and disease‐related disability (IBD Disability Index) were also inversely associated with DPI quartiles (FSS Q4: *β* = −2.47, 95% CI: −3.70 to −1.87; IBD Disability Index Q4: *β* = −7.01, 95% CI: −9.80 to −4.33, all *p* < 0.001), indicating less fatigue and disability in participants adhering to a more phytochemical‐rich diet. Finally, clinical disease activity as measured by the Mayo score was significantly lower in the highest DPI quartile (Q4: *β* = −1.33, 95% CI: −1.75 to −0.90, *p* < 0.001), demonstrating that higher DPI score corresponds with milder disease activity. A reduction greater than 1 point on the Mayo score is generally regarded as clinically relevant in UC trials, underscoring the clinical importance of our findings. These results suggest a robust relationship between greater adherence to a phytochemical‐rich diet and improved psychological well‐being, reduced fatigue, enhanced quality of life, and lower disease activity in this patient population.

**TABLE 5:** Multivariable linear regression of DPI quartiles and psychological well‐being, and clinical outcomes in adults with UC.

Variable	DPI quartile	Crude model	Model 1	Model 2	*p* for trend
PSQI	Q1	Ref	Ref	Ref	
	Q2	−3.37 (−4.02 to −2.72)	−3.39 (−4.04 to −2.74)	−3.37 (−4.01 to −2.72)	
	Q3	−6.46 (−7.09 to −5.82)	−6.44 (−7.08 to −5.80)	−6.45 (−7.08 to −5.82)	
	Q4	−9.89 (−10.56 to −9.23)	−9.90 (−10.57 to −9.23)	−9.85 (−10.55 to −9.33)	< 0.001
PHQ‐9	Q1	Ref	Ref	Ref	
	Q2	−2.95 (−3.52 to −2.38)	−2.96 (−3.53 to −2.39)	−2.96 (−3.52 to −2.40)	
	Q3	−5.47 (−6.03 to −4.92)	−5.46 (−6.02 to −4.90)	−5.46 (−6.00 to −4.91)	
	Q4	−8.57 (−9.15 to −7.99)	−8.57 (−9.15 to −7.98)	−8.25 (−9.01 to −7.9)	< 0.001
GAD‐7	Q1	Ref	Ref	Ref	
	Q2	−1.90 (−2.62 to −1.18)	−1.82 (−2.54 to −1.10)	−1.84 (−2.55 to −1.13)	
	Q3	−4.28 (−4.99 to −3.57)	−4.21 (−4.91 to −3.50)	−4.20 (−4.89 to −3.50)	
	Q4	−6.59 (−7.33 to −5.85)	−6.48 (−7.22 to −5.74)	−6.45 (−7.19 to −5.71)	< 0.001
QOL Assessment SIBDQ	Q1	Ref	Ref	Ref	
	Q2	5.19 (4.78 to 5.60)	5.20 (4.79 to 5.62)	5.19 (4.78 to 5.61)	
	Q3	8.21 (7.81 to 8.62)	8.25 (7.84 to 8.66)	8.26 (7.86 to 8.67)	
	Q4	11.54 (11.12 to 11.96)	11.59 (11.16 to 12.01)	11.54 (11.11 to 11.98)	< 0.001
IBD Disability Index	Q1	Ref	Ref	Ref	
	Q2	−4.14 (−6.76 to −1.52)	−4.09 (−6.73 to −1.46)	−4.11 (−6.75 to −1.47)	
	Q3	−7.57 (−10.14 to −5.01)	−7.47 (−10.06 to −4.89)	−7.41 (−9.99 to −4.84)	
	Q4	−7.50 (−10.18 to −4.81)	−7.37 (−10.09 to −4.66)	−7.01 (−9.8 to −4.33)	< 0.001
Fatigue (FSS)	Q1	Ref	Ref	Ref	
	Q2	−0.70 (−1.63, 0.23)	−0.85 (−1.77, 0.06)	−0.81 (−1.73, 0.12)	
	Q3	−1.89 (−2.80, −0.98)	−1.98 (−2.87, −1.08)	−1.99 (−2.89, −1.08)	
	Q4	−2.66 (−3.61, −1.70)	−2.83 (−3.77, −1.89)	−2.47 (−3.70, −1.87)	0.002
Mayo Score	Q1	Ref	Ref	Ref	
	Q2	−0.27 (−0.68, 0.15)	−0.33 (−0.74, 0.07)	−0.32 (−0.73, 0.09)	
	Q3	−0.89 (−1.29, −0.49)	−0.93 (−1.33, −0.54)	−0.94 (−1.34, −0.54)	
	Q4	−1.28 (−1.70, −0.86)	−1.36 (−1.77, −0.94)	−1.33 (−1.75, −0.90)	0.007

*Note:* Values represent β coefficients and 95% confidence intervals (CIs) from linear regression analyses comparing quartiles of the DPI, with Quartile 1 (Q1) as the reference. *p*‐trend values were calculated using linear regression with DPI as a continuous variable to assess the linear trend of the association with each outcome variable. Crude model is unadjusted. Model 1 adjusts for age, sex, and body mass index (BMI). Model 2 further adjusts for disease duration, supplement use, disease extent, energy intake (kcal/day), physical activity (total MET) and fiber intake (g/day).

Abbreviations: FSS, Fatigue Severity Scale; GAD‐7, Generalized Anxiety Disorder‐7; PHQ‐9, Patient Health Questionnaire‐9; PSQI, Pittsburgh Sleep Quality Index; SIBDQ, Short Inflammatory Bowel Disease Questionnaire.

## 4 Discussion

This study provides evidence for a significant inverse relationship between adherence to a higher score of the DPI and multiple clinical, biochemical, and microbial indicators of disease severity in individuals with UC. Greater adherence to a DPI was associated with a healthier gut microbial profile, reduced markers of systemic and intestinal inflammation, enhanced gut barrier integrity, and improved patient‐reported outcomes, including psychological well‐being, fatigue, sleep quality, and health‐related quality of life. These findings emphasize an association between phytochemical‐rich dietary patterns and reduced UC severity, suggesting their potential as supportive strategies rather than proven adjunctive therapies. Reverse causality cannot be excluded; patients with milder UC may find it easier to adhere to phytochemical‐rich diets. Socioeconomic status, which influences both dietary choices and health outcomes, may partly explain the observed associations.

Our analysis revealed that greater DPI adherence was positively associated with both the F/B ratio and microbial alpha diversity indices. This suggests that a diet rich in plant‐based, phytochemical‐dense foods fosters a more diverse and potentially resilient microbial ecosystem. These results align with prior studies indicating that plant‐rich diets enhance microbial diversity and promote the growth of beneficial taxa known to produce short‐chain fatty acids (SCFAs), such as butyrate and propionate—key microbial metabolites involved in maintaining mucosal integrity and regulating immune function (Sidhu et al. 2023).

Concomitantly, concentrations of SCFAs—including butyrate, acetate, and propionate—were significantly higher among participants with greater DPI adherence (Table 2), suggesting enhanced microbial fermentation and metabolic activity. The higher intake of fermentable fibers, phytochemicals, and polyphenols in phytochemical‐rich diets likely provides substrates that promote the proliferation of SCFA‐producing taxa, particularly Firmicutes, thereby enhancing microbial metabolic output, supporting mucosal integrity, and mitigating intestinal inflammation (Maiuolo et al. 2024; Artis 2008; Yin et al. 2019).

The role of SCFAs in modulating gut integrity was further supported by our observation of lower zonulin levels—an established marker of intestinal permeability (Seethaler et al. 2021)—among individuals with higher DPI scores. Reduced zonulin concentrations suggest improved epithelial barrier function, a key factor in mitigating antigenic translocation and downstream inflammation in UC pathogenesis (Sturgeon and Fasano 2016). These findings are consistent with existing literature demonstrating that plant‐rich dietary interventions can lower zonulin levels and reinforce gut barrier integrity (Del Bo et al. 2021), potentially through the combined effects of butyrate and anti‐inflammatory phytochemicals (Matheus et al. 2023). These findings may have clinical implications for preventing disease flares, particularly among patients with elevated intestinal permeability.

Additionally, a clear inverse association was identified between DPI adherence and multiple inflammatory biomarkers, including FCP, CRP, IL‐6, ESR, and homocysteine. As validated indicators of intestinal and systemic inflammation in inflammatory bowel disease, reductions in these markers highlight the anti‐inflammatory potential of phytochemical‐rich dietary patterns. These findings are consistent with previous research showing that diets high in fiber and anti‐inflammatory nutrients are associated with decreased inflammatory activity and increased SCFA production in individuals with IBD (Kabisch et al. 2025; Yusuf et al. 2022). Mechanistically, SCFAs such as butyrate not only suppress pro‐inflammatory cytokine production and promote regulatory T cell function but also enhance mucosal immune tolerance (Siddiqui and Cresci 2021). At the same time, plant‐based diets reduce the intake of pro‐inflammatory nutrients, including saturated fats and animal proteins, which are commonly implicated in exacerbating intestinal inflammation. Our findings are consistent with those observed for the Mediterranean diet score and the DII, although the DPI more specifically quantifies phytochemical‐rich food intake. Our findings are broadly consistent with Vrdoljak et al. (2020), who reported improved microbial diversity and reduced inflammation with higher adherence to phytochemical‐rich diets. However, methodological differences, including a shorter 61‐item FFQ and smaller sample size, may account for the modest effect sizes reported in their study compared with ours, which utilized a comprehensive 149‐item FFQ and larger cohort.

Participants in the highest DPI quartile exhibited better sleep quality, reduced depression and anxiety, and improved quality of life, potentially due to the systemic anti‐inflammatory effects of phytochemical‐rich diets. Hypothesized mechanisms involving the gut‐brain axis, such as modulation of neuroinflammation or neurotransmitter synthesis via microbial metabolites (Yusuf et al. 2022; Siddiqui and Cresci 2021), are not directly supported by our data, which relied on subjective self‐reported questionnaires (PHQ‐9, GAD‐7) without objective measures (e.g., cortisol levels) or clinical psychiatric confirmation.

Importantly, participants with higher DPI scores were associated with significantly lower Mayo scores, suggesting reduced clinical disease activity. This correlation highlights the potential of phytochemical‐rich dietary adherence to alleviate subjective symptoms and improve objective measures of disease severity. Mechanistically, phytochemical‐rich diets are rich in phytochemicals with anti‐inflammatory and antioxidant properties that may modulate inflammatory pathways in UC (Saleh et al. 2021). These diets are associated with features suggestive of a positive influence on gut microbiota, including a higher abundance of beneficial bacteria that produce SCFAs with anti‐inflammatory effects (Sidhu et al. 2023; Siddiqui and Cresci 2021; van Zonneveld et al. 2024). Additionally, they enhance immune regulation by altering cytokine profiles, reducing pro‐inflammatory cytokines, and improving nutritional status, which is crucial for maintaining remission in UC (Istratescu et al. 2024). Previous studies have shown that diets high in fruits and vegetables correlate with lower disease activity and inflammatory markers, along with improved mental well‐being (Bakhtiari et al. 2024; Pan et al. 2025). Thus, the findings suggest that a phytochemical‐rich dietary approach may lead to meaningful reductions in disease severity in UC, warranting further research to explore causal relationships and mechanisms.

Our findings, demonstrating that higher DPI scores are associated with increased gut microbiota diversity (e.g., Firmicutes/Bacteroidetes ratio, Shannon Index) and reduced inflammatory markers (e.g., CRP, IL‐6, fecal calprotectin) in ulcerative colitis (UC) patients, align partially with previous studies but also exhibit notable differences. For instance, Vrdoljak et al. (2020) reported that adherence to a Mediterranean diet rich in phytochemicals improved gut microbiota composition and reduced inflammation in UC, consistent with our results; however, their smaller effect sizes for CRP reduction may be due to a shorter study duration and less stringent adjustment for dietary fiber intake. Similarly, Godny et al. (2022) found a significant association between phytochemical‐rich diets and reduced fecal calprotectin levels in a smaller UC cohort, though their effect sizes were less pronounced, possibly due to a less comprehensive FFQ that may have underestimated phytochemical intake compared to our 147‐item FFQ (Pan et al. 2025). Discrepancies are not solely due to differences in study populations. For example, our cohort consisted of patients aged 20–60 years with moderate disease activity, whereas Godny et al. included older patients with more severe UC, which may have influenced dietary responsiveness. Furthermore, our adjustment for key confounders—including disease extent and physical activity—in Model 2 likely improved the ability to detect significant associations, unlike studies with more limited adjustment. These findings suggest that proper nutrition may have beneficial effects across a wide spectrum of patients with UC.

This study's strengths include its comprehensive assessment of clinical, microbial, inflammatory, and patient‐reported outcomes, with multivariable adjustments enhancing association validity. Combining objective biomarkers and subjective measures provides a holistic view of disease burden. However, limitations include the cross‐sectional design, precluding causality, and reliance on a validated FFQ, which is subject to recall bias and potential misreporting, affecting DPI accuracy. Although adjusted in multivariable models, dietary/antioxidant supplement use (60.5% of cohort) and higher magnesium/PUFA intake in Q4 may introduce residual confounding. Microbial profiling, limited to 16S rRNA sequencing (Firmicutes/Bacteroidetes ratio, Shannon/Simpson indices), lacks beta diversity, genus‐level taxa, or functional profiling (e.g., PICRUSt, shotgun metagenomics) due to resource constraints. Claims of “beneficial taxa” are inferred from SCFA production and diversity, not direct taxonomic data. Self‐reported psychological outcomes (PHQ‐9, GAD‐7, PSQI) lack objective measures (e.g., cortisol) or clinical diagnoses, limiting mental health conclusions. Reverse causality, where milder UC enables better dietary adherence, is possible. Single‐center recruitment limits generalizability. Future longitudinal studies using metagenomics and metabolomics are recommended to clarify diet–microbiota–inflammation pathways.

## 5 Conclusion

In conclusion, this study indicates that higher DPI scores are associated with improved gut microbial diversity, lower inflammatory burden, enhanced intestinal barrier function, and better clinical and psychological outcomes in patients with ulcerative colitis. However, these findings reflect associations rather than causal relationships, and reverse causality or selection bias may influence results. Longitudinal studies and randomized controlled trials are necessary to confirm these observations.

## Author Contributions

**Yue Zhu:** conceptualization (equal). **Zhihui Lu:** conceptualization (equal), software (equal). **Lingmin Wu:** conceptualization (equal), project administration (equal), writing – review and editing (equal). **Jian Quan:** funding acquisition (equal).

## Ethics Statement

Ethics approval was obtained from the Ethics Committee of the 302 Hospital of China Guizhou Aviation Industry Group (Approval Code: 20221105). Written informed consent was obtained from all participants. All procedures were conducted in accordance with the Declaration of Helsinki.

## Consent

All authors endorse the submission of this manuscript to the journal.

## Conflicts of Interest

The authors declare no conflicts of interest.

## Data Availability

All data generated and analyzed during this study is included in the manuscript. Data supporting the findings of this study are available upon reasonable request from the corresponding author.
